# A High-Stretching, Rapid-Self-Healing, and Printable Composite Hydrogel Based on Poly(Vinyl Alcohol), Nanocellulose, and Sodium Alginate

**DOI:** 10.3390/gels10040258

**Published:** 2024-04-11

**Authors:** Mingyang Li, Yanen Wang, Qinghua Wei, Juan Zhang, Xiaohu Chen, Yalong An

**Affiliations:** 1 Industry Engineering Department, School of Mechanical Engineering, Northwestern Polytechnical University, Xi’an 710072, China; 2 Innovation Center NPU Chongqing, Northwestern Polytechnical University, Chongqing 400000, China

**Keywords:** 3D printing, self-healing property, conductivity, nanomaterials, hydrogel sensor

## Abstract

Hydrogels with excellent flexibility, conductivity, and controllable mechanical properties are the current research hotspots in the field of biomaterial sensors. However, it is difficult for hydrogel sensors to regain their original function after being damaged, which limits their practical applications. Herein, a composite hydrogel (named SPBC) of poly(vinyl alcohol) (PVA)/sodium alginate (SA)/cellulose nanofibers (CNFs)/sodium borate tetrahydrate was synthesized, which has good self-healing, electrical conductivity, and excellent mechanical properties. The SPBC0.3 hydrogel demonstrates rapid self-healing (<30 s) and achieves mechanical properties of 33.92 kPa. Additionally, it exhibits high tensile strain performance (4000%). The abundant internal ions and functional groups of SPBC hydrogels provide support for the good electrical conductivity (0.62 S/cm) and electrical response properties. In addition, the SPBC hydrogel can be attached to surfaces such as fingers and wrists to monitor human movements in real time, and its good rheological property supports three-dimensional (3D) printing molding methods. In summary, this study successfully prepared a self-healing, conductive, printable, and mechanically superior SPBC hydrogel. Its suitability for 3D-printing personalized fabrication and outstanding sensor properties makes it a useful reference for hydrogels in wearable devices and human motion monitoring.

## 1. Introduction

With the prosperity and development of flexible electronics technology, smart wearable devices have been widely used in the fields of human health monitoring, human motion detection, human–machine interfaces, and soft robotics [1,2,3,4]. These devices necessitate sensing materials possessing a level of flexibility and heightened sensitivity to electrical signals. This enables the swift conversion of changes induced by external stimuli, such as pressure [5,6], strain [7,8], temperature [9,10], and acoustic waves [11,12], into electrical signals for efficient recording and transmission. Hydrogel is a flexible material with a three-dimensional network structure [13,14]; its abundance of functional groups and extremely high water content endow it electrical conductivity, which makes it an ideal sensor material for wearable devices. However, the drawbacks of ordinary hydrogels, such as short lifespan, poor tensile rate, and weak mechanical properties, hinder its applications in the field of flexible electronics.

The self-healing hydrogels are capable of repairing damage without external stimuli, extending the life of the material and preserving its original function [15]. It provides support for solving the problem of the lifetime for hydrogel as sensor material. The self-healing principle of self-healing hydrogels all depends on the energy dissipation of their internal dynamic bonds [16,17]. The most common is based on dynamic reversible covalent bonds, such as Schiff bases, acyl hydrazones, borate bonds, and physical hydrogen bonds [18]. Examples include oxidized sodium alginate [19,20,21], poly(vinyl alcohol) [22,23,24], poly(acrylamide) [22,24,25], and chitosan [26,27,28]. In comparison, the formation of borate bonds is relatively straightforward, and these bonds possess a certain degree of adhesiveness, making them suitable for adhering to the body surface as sensors [15,29].

Polyvinyl alcohol (PVA) has attracted widespread interest as a non-toxic, water-soluble, biocompatible, and biodegradable polymeric material [30]. Significantly, the abundant hydroxyl groups in PVA chains can form a large number of boronic acid diol bonds by chemical cross-linking, thus synthesizing hydrogels with excellent self-healing properties [31]. However, excellent self-healing properties require a fast flow of PVA chains in the hydrogel, which may lead to poor mechanical properties of PVA samples with high self-healing performance [32,33]. As a sensor material, poor mechanical properties will limit the application range of sensors [34,35,36]; therefore, enhancing the mechanical properties via nano-reinforcement methods while ensuring high self-healing properties is a feasible approach. Carboxylated nanofibrillated cellulose (CNF) has rich functional groups with excellent mechanical properties [32,37,38], and it can enhance the mechanical properties of hydrogel as a nano-filler [39,40]. Moreover, the hydroxyl groups on the molecular chain can provide a large number of cross-linking points for the composite hydrogel, so as to further entangle the internal network structure and enhance the energy dissipation capacity of the hydrogel network [41,42,43]. On the other hand, wearable devices have a wide range of application scenarios, and it is difficult to fabricate hydrogels from 2D to 3D structures by traditional casting methods, but 3D printing can support the fabrication of 3D flexible sensors through layer-by-layer deposition molding methods [44,45,46]. Therefore, it is of great significance to develop composite hydrogels with self-healing, electrical conductivity, excellent mechanical properties, and printability.

In this work, we prepared a printable self-healing hydrogel SPBC, which is based on PVA/SA/CNF matrix material cross-linked with sodium tetraborate. The hydrogel has good self-healing properties (<30 s) and mechanical properties (33.92 kPa) and an excellent tensile ratio (>4000%). In addition, the excellent electrical conductivity (0.62 S/cm) of the SPBC hydrogel enables it to be used as a motion sensor for monitoring human movements (fingers, wrists, etc.).

## 2. Results and Discussion

### 2.1. Synthesis and Characterization of Hydrogel

**Figure 1:**
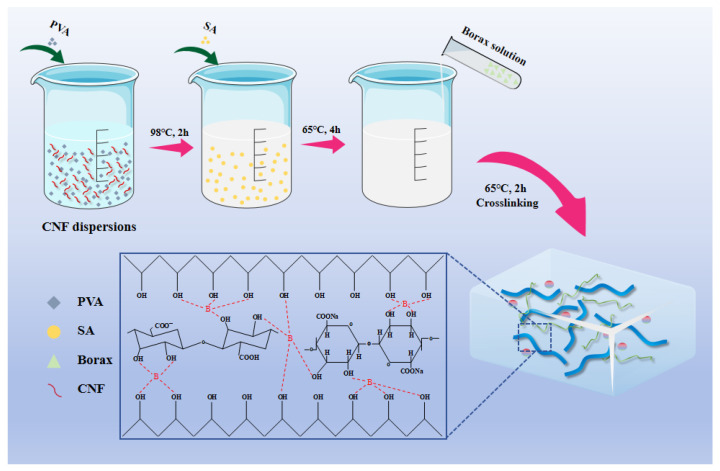
Schematic diagram of SPBC hydrogel synthesis principle.

The SPBC hydrogels were synthesized using a one-pot method, as illustrated in Figure 1. PVA and SA in specified amounts were added to the CNF suspension and were heated at 98 °C and 65 °C for 2 h separately to form precursor solutions. Subsequently, a specific volume of a borax solution (1.5 wt%) was introduced into the precursor solutions, and the mixture was kept at a constant temperature for 2 h to ensure complete cross-linking and uniform hydrogel formation.

**Figure 2:**
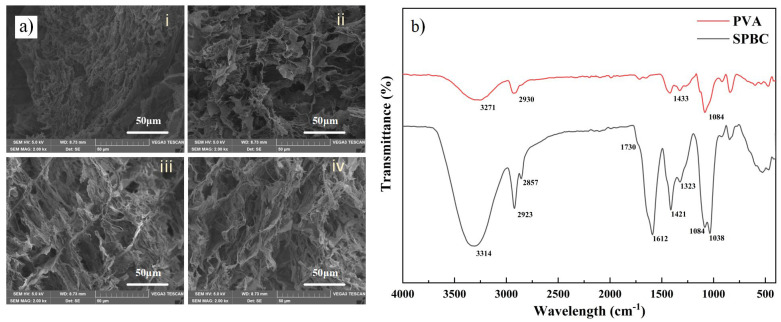
The characterization of the internal structure of composite hydrogels. (**a**) The SEM image of the hydrogel samples (i: SPB, ii: SPBC_0.1_, iii: SPBC_0.2_, iv: SPBC_0.3_). (**b**) The FTIR spectrogram of the hydrogel sample.

The micromorphology of the lyophilized hydrogels was observed by SEM. As shown in Figure 2a, all hydrogel samples exhibit porous structure. Compared with the larger pore structure of the SPBC hydrogel, the original SPB hydrogel showed the smallest pore structure. This difference arises from the sufficient intermolecular cross-linking induced by an appropriate amount of the borax solution in the SPB hydrogel. The introduction of CNF brings a significant number of hydroxyl groups that react with the borax solution, leading to a lower cross-linking degree in PVA and the formation of larger pore structures. Furthermore, with the increase in CNF content, more chemical bonds and hydrogen bonds are formed in the hydrogel, which is conducive to the enlargement of pores.

The FTIR test was performed on the SPBC hydrogel to analyze the interactions between the materials in the hydrogel. As shown in Figure 2b, the hydrogel exhibits a broad and strong peak at 3000–3700 cm^−1^ as the characteristic peak of -OH [47,48]. Compared with the -OH characteristic peak of the hydrogel moving to 3314 cm^−1^, the hydroxyl characteristic peak of pure PVA was at 3271 cm^−1^. This indicates that the addition of SA and CNF not only increases -OH, but also forms a large number of hydrogen bonds between the molecules [49,50]. At 1612 cm^−1^ and 1730 cm^−1^, it is the carboxyl/sodium carboxylate absorption peaks [51], indicating the successful doping of sodium alginate and CNF. The absorption peak at 1084 cm^−1^ is attributed to the stretching vibration of the C-O single bond [52]. At 1421 cm-1 and 1038 cm^−1^, the characteristic peaks are attributed to the B-O-C asymmetric stretching vibration in the SPBC hydrogel [53], which also indicates that there is a boron diol bond formation in the hydrogel and an interaction between PVA and borax.

### 2.2. Mechanical Properties of SPBC Hydrogels

**Figure 3:**
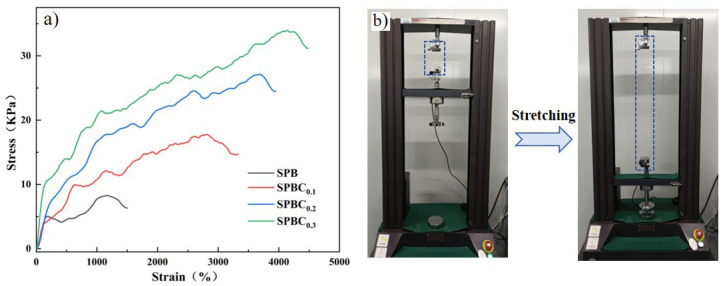
Mechanical testing of SPBC hydrogels. (**a**) The schematic diagram of SPBC hydrogel synthesis. (**b**) The process of SPBC hydrogel stretching.

The mechanical test of the hydrogel was performed using a universal testing machine to further analyze the influence of nanofibrillar cellulose content on the strength and toughness of SPBC hydrogel. As shown in Figure 3a, it can be obviously seen that compared with SPB hydrogel without nanomaterials, SPBC hydrogel containing CNF has significantly better mechanical properties and a superior tensile ratio. The original SPB hydrogels had a tensile stress of 8.27 kPa and a tensile ratio of 1178%, both of which are much smaller than the SPBC hydrogel. In the stress–strain curve, the tensile stress of SPBC0.1 is 17.7 kPa with a tensile strain of 2814%. With the increase in CNF content, the mechanical properties of the composite hydrogel improve. The tensile stress of SPBC0.2 increases to 27.1 kPa, with a tensile strain of 3689%. With the increase in CNF concentration from 0.1 wt% to 0.3 wt%, the tensile stress (33.92 kPa) of the SPBC0.3 hydrogel is three times higher than that of SPB hydrogel, and the tensile ratio is increased by 2.5 times, reaching 4148%. The obvious improvement in tensile properties indicates that the added CNF not only plays the role of nano-enhancement, but also interweaves the nanofiber chains, with a large number of hydroxyl groups on the chains interlinked with the hydroxyl groups of PVA and SA, forming a large number of borate bonds in the presence of the borax solution. Therefore, it leads to the interweaving of various molecular networks, thus enhancing the mechanical properties of SPBC hydrogels. Under a certain proportion of the borax solution, PVA and SA in SPB hydrogel are completely cross-linked with sodium tetraborate, resulting in the brittle nature of SPB hydrogel. During external stretching, the internal network of the hydrogel is unable to dissipate the absorbed energy promptly. This limitation results in unsatisfactory mechanical properties and tensile ratios for the hydrogel. Compared with the original SPB hydrogel, the addition of CNF not only participates in the chemical reaction in the hydrogel, but also the strong nanofiber chains act as a bridge to connect the interior firmly, forming more borate and hydrogen bonds with PVA and SA, increasing the complexity of the interior of the hydrogel and improving the mechanical properties of the hydrogel. The CNF dispersion was in a colloidal state, and the precursor solution of SPBC hydrogel with 0.4 wt% CNF is too thick, resulting in difficulties in preparing homogeneous hydrogels. Therefore, SPBC0.3, which exhibits excellent mechanical properties, was selected for subsequent experiments.

### 2.3. Self-Healing Performance

**Figure 4:**
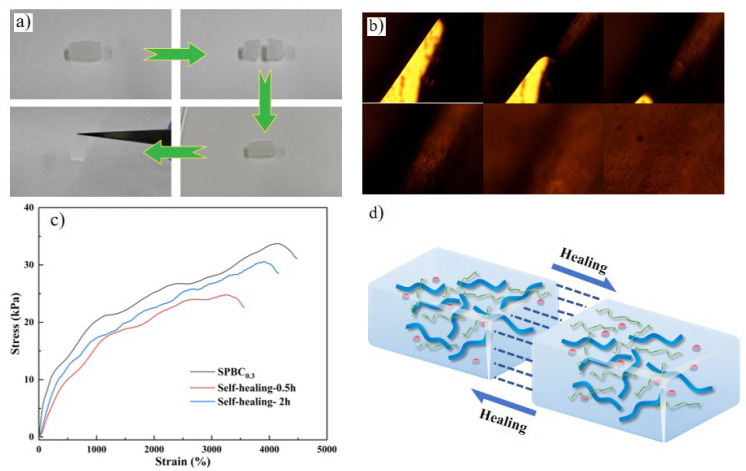
The self-healing behavior of SPBC performance. (**a**) The self-healing process of hydrogels: cut, contact, healing. (**b**) The self-healing process of hydrogels under an optical microscope (0 s, 10 s, 20 s, 30 s, 30 min, 1 h). (**c**) Tensile properties of self-healing SPBC hydrogels. (**d**) A schematic representation of the SPBC hydrogel’s self-healing properties.

The self-healing properties of SPBC hydrogels were analyzed by macroscopic self-healing experiments and tensile tests. As shown in Figure 4a, the cuboid samples were cut in half, and the cut surfaces of the samples were brought into contact with each other at room temperature. The hydrogel’s self-healing behavior was observed under no external force. After 5 min of sample contact, the boundary at the interface becomes blurred. Although there are some faint traces, it is evident that the two hydrogel samples have been connected as a body. Using tweezers to lift the healed hydrogel samples, it is found that the self-healed samples support their own weight without fracturing. The self-healing phenomenon is due to the reaction of sodium tetraborate with the -OH groups on the molecular chains of PVA, SA, and CNF, resulting in the formation of dynamic and reversible borate ester bonds [54,55]. Besides the borate bonds, a large number of hydrogen bonds are also formed between the polymer molecular chains, which greatly facilitates the reconstruction of chemical bonds in hydrogels. A significant number of borate and hydrogen bonds are broken when SPBC hydrogels are cut. The sample cut surfaces are in contact with each other. Although there is no external force, as the molecules are also in constant motion, a large number of borate and hydrogen bonds are reorganized on the cut surfaces, which leads to the self-healing behavior of the SPBC hydrogel. As shown in Figure 4b, the self-healing process of the hydrogel was observed using a vertical metallographic microscope. It can be observed that initially, when the fracture surfaces are just in contact, the gaps are clearly visible. However, with the variation in contact time, at 10 s and 20 s, the fractured hydrogel has begun to heal, and the fracture surface gradually becomes blurred. At 30 s, the fractured hydrogel is fully reconnected, and there is no obvious gap on the contact surface. After 30 min, with the movement of water molecules and PVA polymer chains on the contact surface, borate bonds and hydrogen bonds are re-established, completing the healing process substantially. After 1 h, the traces of the fracture surface have completely disappeared. Further tensile tests were conducted on the healed hydrogel, as shown in Figure 4c, revealing an improvement in mechanical performance recovery with the increase in healing time. After 30 min of healing, the tensile stress of the sample (24 kPa) is only 73% of the original sample, while after 2 h of healing, the tensile stress of the sample (31 kPa) recovers to 91% of the original sample. Furthermore, we found that the SPBC hydrogel samples have excellent self-healing properties and are able to heal rapidly in the absence of external forces.

### 2.4. Rheological Behavior

**Figure 5:**
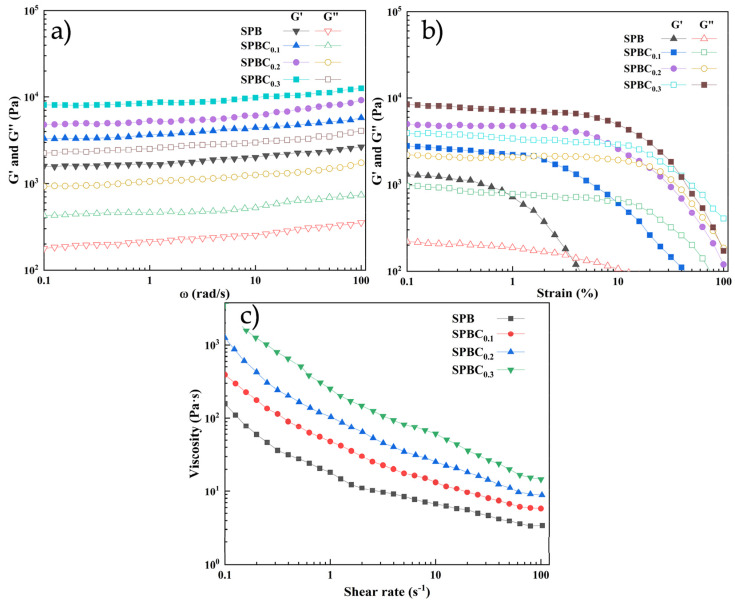
Rheological properties of SPBC hydrogels. (**a**) Perform frequency scans on each sample, conducted at a 0.5% strain, with a scanning range of 0.1~100 Hz. (**b**) Amplitude scans were carried out for each sample at a scanning frequency of 6.28/rad with a strain range of 0.1 to 100%. (**c**) The shear rate table for SPBC hydrogels. G’ is the energy storage modulus and G’’ is the loss modulus.

Rheological tests can reflect the viscoelastic behavior of SPBC hydrogel materials. Frequency scans were performed on all hydrogel samples in the range of 0.1 to 100 Hz, as shown in Figure 5a. All hydrogel samples significantly show a higher energy storage modulus (G’) rather than a loss modulus (G’’), which indicates that the hydrogel samples are able to maintain a stable gel state over a large frequency range. The energy storage modulus varies from hydrogel to hydrogel, and the G’ of SPBC hydrogels are all higher than that of SPB hydrogels, and the G’ increases with the increase in CNF content, which is consistent with the mechanical property test. This is due to the introduction of CNF providing more cross-linking points for borate and hydrogen bonds. In addition, amplitude scans were conducted on each hydrogel sample in the range of 0.1% to 100%. The results are shown in Figure 5b; in the low-strain-region hydrogel samples, the very beginning of G’ is much larger than G’’, which indicates the gel property, but with the gradual increase in the strain, regarding each gel, respectively, at different strain positions, the beginning of G’’ is larger than G’, which shows the sol–gel state. Since there are a large number of hydrogen bonds in the hydrogel, it is able to resist a certain degree of strain, but as the strain gradually increases, a large number of hydrogen bonds are broken, resulting in a sharp decrease in G’ and thus a morphology transition. The characteristics of SPBC hydrogel gel–sol morphology conversion support the personalized manufacturing of 3D printers.

The graph of the shear rate of each hydrogel sample is shown in Figure 5c, and the viscosity decreases gradually as the shear rate increases, which allows the SPBC hydrogel to be smoothly extruded from the printing needle through the syringe under a certain pressure.

**Figure 6:**
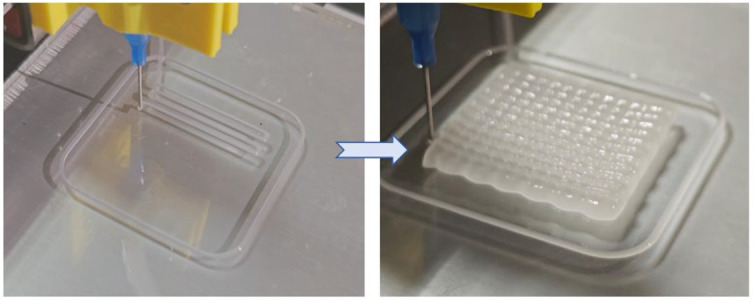
The process of 3D printing on SPBC hydrogels.

The hydrogel precursor solution was loaded into the syringe and the appropriate amount of the borax solution was dripped in for cross-linking, and the syringe was held at 65 °C for 2 h to ensure that the hydrogel in the syringe was fully cross-linked. We used the open-source Repetier-Host v2.3.2 for path planning followed by printing. The printing process is shown in Figure 6. The diameter of the needle tip of the syringe is 0.6 mm, with a moving speed of 8 mm per second, and the sample size is 30 × 30 × 5 mm. The SPBC hydrogel is extruded from the needle, and its rheological properties enable the extruded hydrogel to achieve self-support. The hydrogel deposits layer by layer, ultimately forming a three-dimensional structure.

### 2.5. Electrical Properties

**Figure 7:**
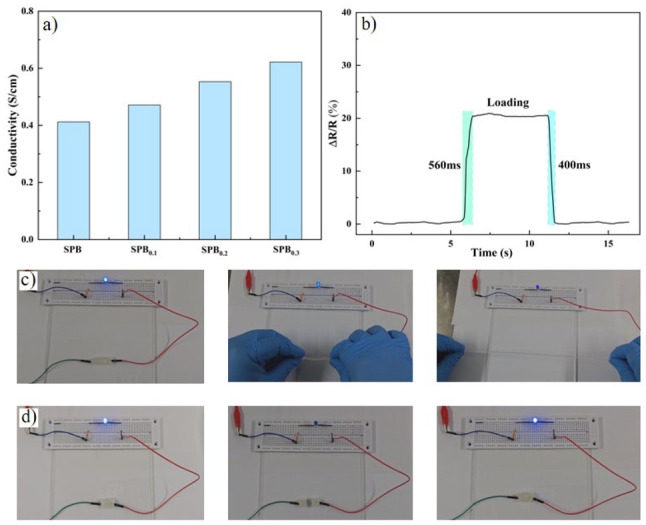
Conductivity testing of SPBC hydrogels. (**a**) Conductivity of SPBC hydrogels with different CNF contents. (**b**) Testing responsiveness to sudden loading of hydrogels. (**c**) The relationship between the elongation rate of SPBC hydrogels and the brightness variation of the bulb in the circuit. (**d**) The self-healing and electrical recovery properties of hydrogels.

The electrical conductivity of SPBC hydrogels was further characterized by designing circuit experiments and a digital measuring instrument. Depending on the synthesis principle of SPBC hydrogel, the hydrogel possesses a large amount of water and various free ions, which endows SPBC hydrogel with the ability to conduct electricity. In addition to the free ions such as Na^+^, H^+^, and B(OH)4^−^ providing conductivity, a large number of functional groups (-COO^−^) in the gel also contribute to the conductivity of the hydrogel [33,56]. In Figure 7a, the conductivity of different hydrogel samples is shown, and it is found that the conductivity of SPBC hydrogel gradually enhances with the increase in CNF content. This phenomenon is attributed to the introduction of carboxyl groups during the TEMPO-mediated preparation of nanofibrillated cellulose, which simultaneously introduces a portion of sodium carboxylate [57,58]. Consequently, an increase in the concentration of CNF results in an increase in sodium ions in the hydrogel, thereby enhancing the electronic properties of SPBC hydrogel. Additionally, carboxylated nanofibrillated cellulose exhibits electronegativity [59,60], which further enhances the hydrogel’s conductivity to a certain extent. The hydrogel samples were abruptly subjected to 30% strain for 5 s, and then the strain recovered to 0%. The electrical response capability of SPBC hydrogel was tested using a simulated stepwise electrical signal. The results are depicted in Figure 7b, where the rate of change in resistance (∆*R*/*R*) of the SPBC hydrogel suddenly increases, remains stable for 5 s, and then swiftly returns to the initial value. This pattern demonstrates the excellent electrical response (560 ms) and electrical recovery (400 ms) of SPBC hydrogel. Additionally, circuit experiments were designed. In Figure 7c, it can be observed that the brightness of the blue LED decreases with the increase in stretching length. This is because, as the hydrogel is stretched, its cross-sectional area gradually decreases, leading to an increase in the hydrogel’s resistance, resulting in the dimming of the blue LED light. As depicted in Figure 7d, cutting the hydrogel in the circuit results in the blue LED turning off, and the LED immediately lights up upon the sections coming into contact with each other. Therefore, SPBC hydrogel is a potential flexible sensor material with excellent electrical properties and fast responsiveness to strain.

### 2.6. Sensing Performances

**Figure 8:**
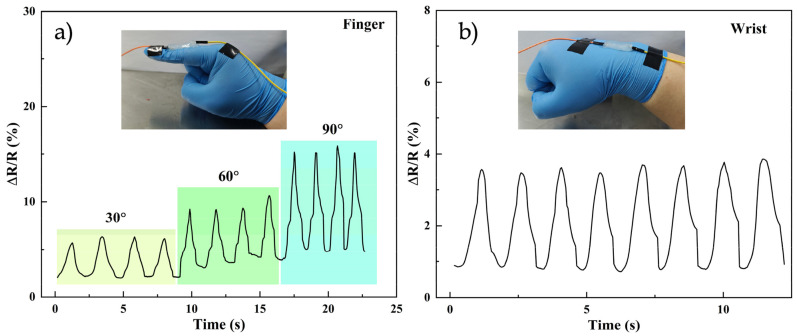
SPBC hydrogels were used as monitoring sensors for electrical experiments. (**a**) Finger. (**b**) Wrist.

According to the excellent electrical and mechanical properties of SPBC hydrogel, the potential application of the hydrogel as a sensor should be further explored. The SPBC0.3 hydrogel was cut into a rectangular shape, connected to wires at both ends, and attached to body surfaces such as fingers, wrists, etc., for behavioral monitoring of human movements. As shown in Figure 8a, the hydrogel was placed on the index finger, and the two ends were connected to a digital measuring instrument, which measures the resistance value of the hydrogel to record the movement of the index finger. The index finger moved from a horizontal position to a 30° angle position, then returned to the original position, repeating the action several times. Subsequently, angles of 60° and 90° were tested. The rate of change of the resistance value is shown in Figure 8a. As the fingers were bent at 30°, 60°, and 90°, the rates of change in the resistance values are 6.8%, 10.2%, and 15.6%, respectively. The resistance values returned to the initial position after each return to the horizontal position, thereby confirming the high sensitivity of SPBC hydrogel. A test was also performed on the wrist and the relative resistance change was monitored as the wrist joint was flexed, as shown in Figure 8b. Different bending angles result in different hydrogel strains and different resistance changes. It can not only monitor the finger and wrist area, but also monitor the knee, elbow, and other parts. By collecting and studying the data from different sports scenes, it can change the electrical signals and obtain different movement patterns. Therefore, SPBC hydrogels have great potential in motion sensors.

## 3. Conclusions

In summary, we have successfully developed a nano-enhanced SPBC hydrogel. This composite hydrogel is synthesized through the reaction of PVA, SA, and CNF with sodium tetraborate. The incorporation of CNF nanomaterials endows the SPBC hydrogel with outstanding performance, such as a tensile strength of 33.92 kPa, a tensile strain of up to 4000%, and a conductivity of 0.62 S/cm, enabling rapid self-healing and support for 3D printing. Importantly, severed samples can self-heal within 30 s without external intervention, attributed to the presence of abundant dynamically reversible borate and hydrogen bonds within the SPBC hydrogel, enhancing its self-healing properties. Additionally, the hydrogel’s rich ions and functional groups also support its conductivity. By monitoring the resistance changes in the SPBC hydrogel, real-time monitoring of finger and wrist movements is feasible, making it highly suitable for strain sensors. Overall, the SPBC hydrogel exhibits excellent performance and holds potential for applications in human health monitoring.

## 4. Materials and Methods

### 4.1. Materials

The carboxycellulose nanofibers (CNFs, 0.8 wt%) were procured from Qihong Bio-technology Co., Ltd. (Guilin, China). Poly(vinyl alcohol) (PVA, 1799, Mw≈74,800) and sodium alginate (SA, AR) were obtained from McLean Biochemical Technology Co., Ltd. (Shanghai, China). Sodium borate decahydrate (Borax, AR) was acquired from Aladdin Biochemical Technology Co., Ltd. (Shanghai, China). Deionized water was supplied by a laboratory water purifier, and all materials were used directly without further processing.

### 4.2. Synthesis of SPBC Hydrogels

Adequate amounts of 1799 PVA and CNF dispersions were added to deionized water and stirred for 2 h at 98 °C in a water bath to prepare a homogeneous mixed solution. The PVA concentration in this solution is 8 wt%. A small amount of deionized water was added to control the CNF concentration at 0.1 wt%, 0.3 wt%, and 0.5 wt%. Subsequently, 0.4 g of SA powder was added to the mixed solution and stirred for 4 h at 65 °C in a water bath until the SA powder completely dissolved, with a mass fraction of 1 wt%. Additionally, a borax solution with a concentration of 1.5 wt% was prepared at room temperature. Finally, 15 mL of the borax solution was added to the mixed solution to induce cross-linking, and the mixture was held at 65 °C for 2 h to sufficiently cross-link the materials, resulting in the formation of the SPBC hydrogel.

### 4.3. Structure Characterization

The composition of the composite hydrogels was analyzed by Fourier-transform infrared spectroscopy (FT-IR). The equipment used was a Bruker Alpha II (Bruker, Salbruken, Germany), and the composite hydrogels were tested using a diamond crystal attachment under the attenuated total reflection mode (ATR). The test wave number range was 400–4000 cm^−1^ with a resolution of 4 cm^−1^. Due to the principles and characteristics of the ATR mode, detection could be performed by direct contact with the composite hydrogel containing water.

An analysis of pores and micromorphology of hydrogel samples was performed by a scanning electron microscope, VEGA3 (TESCAN, Prague, Czechoslovakia). The hydrogel samples were frozen at −80 °C, and the frozen samples were freeze-dried for 24 h using the freeze-dryer TF-FD27 type (TianFeng, Shanghai, China). In addition, the lyophilized samples were broken in the middle and the fractured surface of the samples was observed by gold spraying.

### 4.4. Rheological Characterization

The rheological properties of hydrogel samples were analyzed using a NETZSCH Rheometer Prime Ultra+ (Kinexus, Selb, Germany). A plate with a diameter of 20 mm was utilized for all samples and tests. The sample underwent frequency scanning at a constant 0.5% strain within a frequency range of 0.1~100 Hz. Amplitude scans were conducted on the samples at a constant frequency of 1 Hz for a strain range of 0.1 to 100%. The energy storage modulus is denoted as G’, and the dissipation modulus is denoted as G”.

### 4.5. Mechanical Tests

The mechanical properties of the composite hydrogels were characterized using a universal material testing machine Instron 5943 (Instron, Norwood, MA, USA). Hydrogel samples were cut into cubes with dimensions of L: 40 mm, W: 10 mm, H: 5 mm. The samples were securely fixed at both ends using a fixture and subjected to tensile testing at a speed of 100 mm/min. Here, L represents the sample length, W represents the sample width, and H represents the sample height.

### 4.6. Self-Healing Properties

Hydrogel samples were cut in the middle, and the broken hydrogels were brought into contact with each other to observe the self-healing behavior. To evaluate the self-healing capabilities, experiments were conducted at room temperature without external interference, and the fracture sites were examined using a 9XB-PC (Shanghai Optical, Shanghai, China) vertical metallographic microscope.

### 4.7. Three-Dimensional Printing of Hydrogels

The SPBC hydrogel printing was achieved in this study using an extrusion 3D printer developed independently in the laboratory. The SPBC hydrogel precursor solution was loaded into the printer syringe. The hydrogel printing process was carried out through the open-source software Repetier-Host v.2.3.2 which was utilized for path planning and extrusion control. The syringe had a needle diameter of 0.6 mm, and the moving speed was set at 8 mm/s.

### 4.8. Conductivity Performance

The hydrogel samples were cut into cubes and a probe of a digital measuring instrument was fixed at each end of the sample to study the conductive behavior of the composite hydrogel. The conductivity of the hydrogels exhibited variations under different strains. A digital measuring instrument, DMM6500 (Keithley, Cleveland, OH, USA), was employed to record the changes in electrical resistance during hydrogel strain and to export the data. The relative change in the resistance value was calculated using the formula *ΔR/R0 = (R−R_0_)/R_0_*, where *R_0_* represents the resistance under no strain, and *R* represents the resistance under strain. For sensor testing, a rectangular hydrogel sample was positioned on the surface of the finger or wrist, with wires connected at both ends, and human behavior was monitored using a digital measuring device.

## Data Availability

The raw data supporting the conclusions of this article will be made available by the authors on request.
